# The Fiftieth Memorial Celebration

**Published:** 1895-01

**Authors:** 


### Editorial.

### THE FIFTIETH MEMORIAL CELEBRATION.

When the proposition was originally made by Dr. J. D. Thomas, before the Odontological Society of Pennsylvania, that a celebration of the discovery of anæsthesia should be made, it was with no idea that it would extend beyond the confines of Philadelphia and would be, as intended, a local celebration by a local society. The mere announcement of the fact, or what we prefer to believe, a simultaneous impression, led to an immediate effort in various directions to celebrate this event in other localities. That this would deprive it of all educational value seemed clearly evident, and an effort was made, very properly, to nationalize it.

When the American Dental Association met last August at Old Point Comfort, Virginia, the question was brought up by Dr. Thomas, and, after due consideration, the matter was placed in the hands of a committee with power to act. The feeling throughout that large meeting was one of enthusiasm, the only drawback was the question, "Will the dentists of the country, or any considerable number of them, feel sufficient interest to make the journey to Hartford, Connecticut, in December, for no other place was regarded as appropriate for this celebration but the city where Wells lived and worked out this great problem. It was finally decided that the dental profession were in duty bound to celebrate the semi-centennial of the discovery of anæsthesia; that the time had arrived to proclaim to the world where, and by whom, this discovery was made.

There was no question raised as to the propriety of holding this, nor was there any contention as to place, as Hartford, Connecticut, was regarded as the one foreordained for it; but when the delegation from that city was consulted, it was found that they specially desired to have a local celebration of the event, and further, they were unanimously of the opinion that the national celebration should be held in some one of the large cities. The force of these arguments, coming from the city most interested, left no other course for the Association than to decide upon the city in which the celebration should be held, and without any dissenting voice Philadelphia was selected.

The committee, under the leadership of their energetic chairman Dr. J. D. Thomas, proceeded at once to perfect arrangements, and issued a circular, published in the November number of this journal and elsewhere, calling upon the profession to respond generously to this action of the American Dental Association.

The 11th of December, 1894, arrived, and with it came numbers to Philadelphia from fourteen States of the country, imbued with a spirit of professional pride, a gratifying proof of the deep interest felt in making this occasion an impressive one. For the details of this Memorial Celebration we must refer our readers to the report on another page.

It was one of those periods that come to the life of men, professions, and peoples when "New occasions teach new duties," and we believe that the meeting performed, unexpectedly, an educational part in that it impressed all present with the fact that more than the discovery of anæsthesia, more than the honor due to Wells, more than a mere celebration of a great event in the history of the world, was the fact that this marked a dividing line between the older conception of duty and that broader and more generous idea of what should constitute professional life. It breathed into the souls of those present the finer thought that selfishness must be relegated to its proper level if the profession would develop the highest good.

The admirable addresses delivered in the afternoon struck the key-note of this idea. They properly eulogized Wells and his work, not forgetting Morton, Jackson, and the host of allied co-workers. The underlying conception seemed, however, to be that from this germ-thought of the man there arose a system of relief from pain, of benefit to the entire sentient life of the world, and that this glorified the century.

We have but one criticism to make, and it almost seems ungenerous to give it expression, yet the truth of history requires it. In the address of Professor Fillebrown the name of Simpson was given prominence as though he were the discoverer of chloroform. He was simply an active agent in promoting its use in his profession. For this he is entitled to great honor, but as Dr. B. W. Richardson expresses it (*Longman's Magazine*), "By one of those extraordinary frolics of fortune, never explainable, Simpson, seventh on the list of expositors of anæsthesia, leaped into the first place, and for almost a generation was believed to be the actual author of the process." The discovery of chloroform belongs to Guthrie, or, as Dr. Garretson properly places it, Guthrie, Liebig, Soubeiran, and in the order named.

The banquet of the evening was something more than a mere gastronomic performance. The men assembled were not there simply to eat and make merry, but all apparently felt the deep responsibility of the occasion. Fifty years was behind the world of surgery in the use of anæsthetics, and now had arrived the crucial hour, the closing of a half-century of experience in which the work was to be analyzed and the corner-stone of the monument to the honor of the discoverer was to be laid, and that so firmly that succeeding generations could point to this period without any misgivings and say truly, that to Wells belongs the honor of this discovery, and that to him the world is indebted for relief from the agony of the surgeon's knife.

The occasion was more than this. To the writer it seemed as though dentistry had received a new birth. The old was being cast aside, and in newness of spirit it had suddenly become a more glorified profession. It had passed through its day of trials, and now, on the soil dedicated to historic memories, it had cast off the old things, and, in harmony with the professions, its mission for the future was assured. Here theology, medicine, law, letters, and dentistry met on common ground, and over the grave of the great immortal was cemented a higher bond; and it only now remains for dentistry at large to absorb the lesson, hold it in reverential memory, and to practise its regenerated life.

